# Giant laryngeal neuroendocrine neoplasm causing airway obstruction: A case report and literature review

**DOI:** 10.1097/MD.0000000000038382

**Published:** 2024-05-31

**Authors:** Yaqi Wang, Longqing Ding, Jiahui Liu, Ying Guo, Yisong Yao, Xi Chen, Yakui Mou, Xicheng Song

**Affiliations:** a Shandong Provincial Clinical Research Center for Otorhinolaryngologic Diseases, Yantai Yuhuangding Hospital, Qingdao University, Yantai, P. R. China; b Department of Otolaryngology, Head and Neck Surgery, Yantai Yuhuangding Hospital, Qingdao University, Yantai, P. R. China; c School of Clinical Medicine, Shandong Second Medical University, Weifang, P. R. China; d Department of Otorhinolaryngology, The First Affiliated Hospital of Shandong First Medical University & Shandong Provincial Qianfoshan Hospital, Ji’nan, P. R. China.

**Keywords:** fiberoptic laryngoscopy, immunohistochemistry, laryngeal neuroendocrine neoplasm, treatment

## Abstract

**Rationale:**

Laryngeal neuroendocrine neoplasm (NEN) is a rare and heterogeneous disease that originates from neuroendocrine cells. It mainly occurs in middle-aged and elderly men. Due to the lack of specific clinical and imaging manifestations, diagnosis and treatment of the disease pose a challenge. Therefore, a consensus on the diagnosis and treatment of the disease is necessary. By discussing this case, we will be able to gain further insight into laryngeal NEN and will be able to provide some recommendations for the future management of this rare disease.

**Patient concerns:**

A 67-year-old man was admitted to our department with a history of sore throat and dyspnea. After admission, the patient experienced acute airway obstruction and experienced an emergency bedside tracheotomy.

**Diagnoses:**

Flexible fiberoptic laryngoscopy and enhanced CT showed a cauliflower-like mass in the left supraglottic region and obstructed most of the laryngeal cavity. We biopsied the mass, and the pathology showed a poorly differentiated adenocarcinoma.

**Interventions:**

A horizontal hemilaryngectomy and left neck dissection were performed. At 4 weeks after the operation, the patient underwent chemotherapy and radical radiotherapy.

**Outcomes:**

After a 1-year postoperative follow-up, the patient recovered well and showed no signs of recurrence.

**Lessons:**

Laryngeal neuroendocrine neoplasm is very rare, early diagnosis remains difficult. Radical surgery combined with postoperative chemoradiotherapy is currently the most appropriate treatment.

## 1. Introduction

Laryngeal neuroendocrine neoplasms (NENs) are extremely rare tumors in the larynx, constituting <1% of laryngeal neoplasms. However, they are the second most common type after laryngeal squamous cell carcinoma.^[1]^ NENs are most frequently found in the gastrointestinal tract, followed by the lungs, and are infrequent in the head and neck.^[2]^ The first case of a laryngeal neuroendocrine tumor (NET) was reported by Blanchard et al in 1955, and to date, around 500 cases have been documented.^[3,4]^ According to the 2022 WHO classification of head and neck tumors, the laryngeal NEN is classified into 2 distinct categories: well-differentiated NET G1, G2, G3, and poorly differentiated neuroendocrine carcinoma (NEC), which are further divided into subtypes: small cell NEC (SmCNEC) and large cell NEC (LCNEC).^[5]^ Treatment for laryngeal NENs is not yet standardized due to their rarity. According to a meta-analysis of 436 cases of laryngeal NENs, typical carcinoids can be treated by local excision alone, atypical carcinoids should be managed through radical resection and elective neck dissection if conditions permit, as they seem to be insufficiently sensitive to radiotherapy, and poorly differentiated NEC (G3) seem to benefit most from chemoradiotherapy.^[6]^ In this paper, we reported a case of laryngeal NEN, which may provide some insight into the diagnosis and treatment of this disease.

## 2. Case presentation

A 67-year-old man was admitted to our department with a history of sore throat for the past 3 months and dyspnea for the past week. He had taken oral antibiotics, but the results were not satisfactory. The patient had a history of smoking and drinking, but stopped both habits 7 years ago. Additionally, it is important to note that his mother passed away due to lung cancer. No abnormalities were detected during the physical examination.

**Figure 1.**
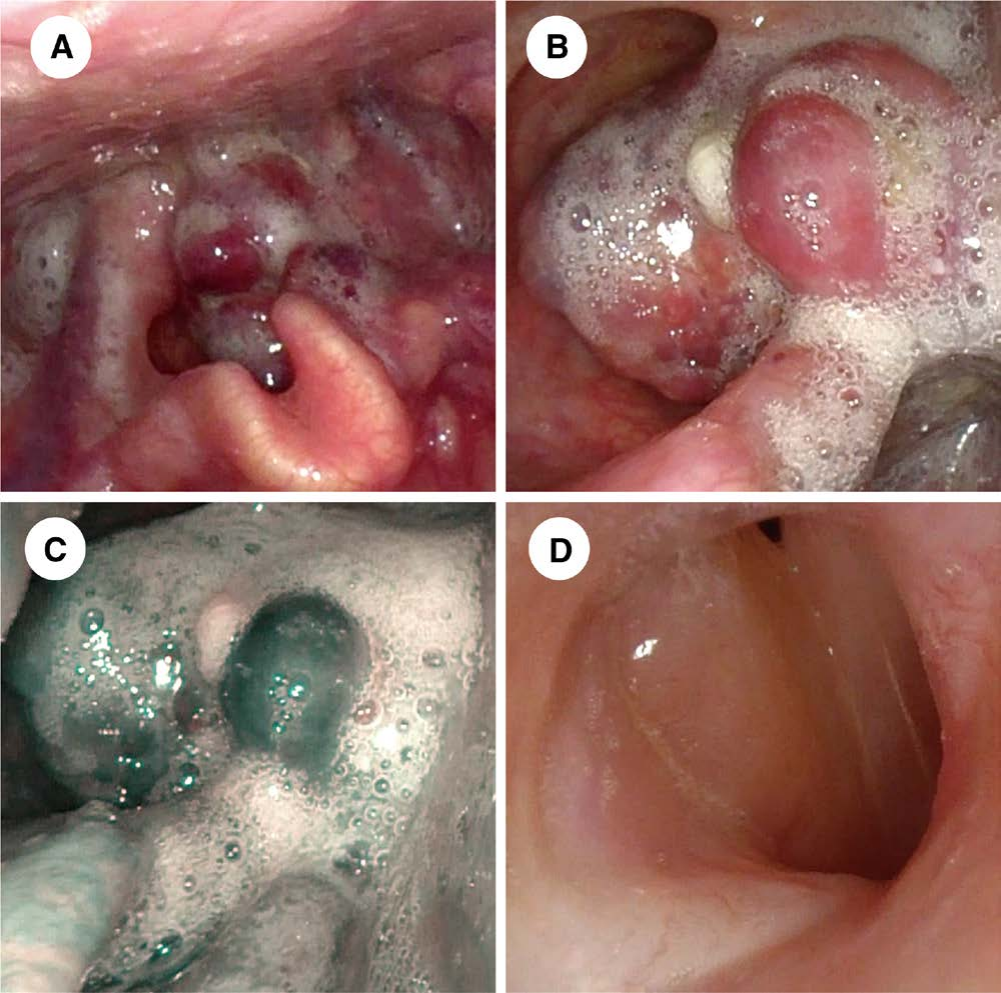
(A and B) Preoperative flexible fiberoptic laryngoscopy showed a cauliflower-like mass originating from the left aryepiglottic fold and obstructing most of the laryngeal cavity and (C) no abnormalities were detected in NBI mode. (D) A 1-yr postoperative follow-up flexible laryngoscopy showed smooth laryngeal mucosa with edema and no signs of recurrence. NBI = narrow-band imaging.

**Figure 2.**
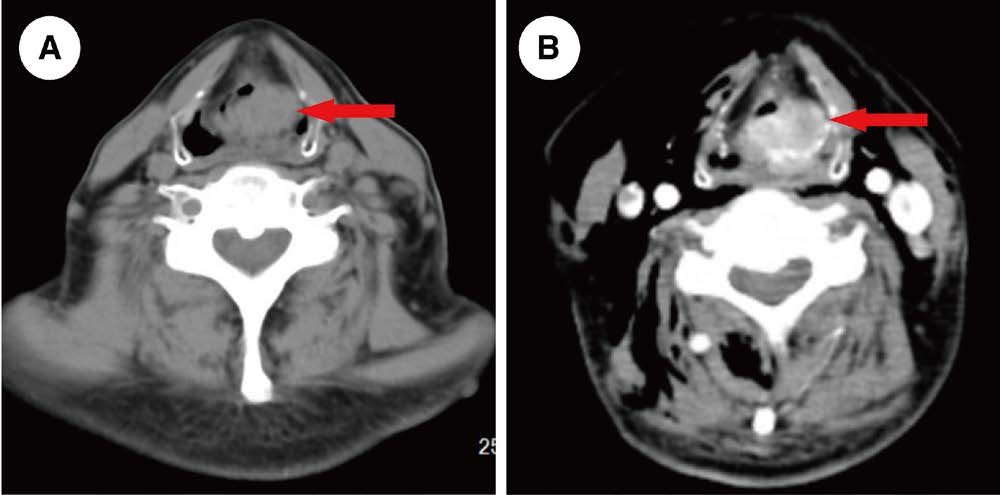
Enhanced CT showed a shadow of soft tissue density in left supraglottic region with an indistinct border with surrounding tissues and uneven enhancement (red arrow). CT = computed tomography.

Flexible fiberoptic laryngoscopy revealed a cauliflower-like mass that originated from the left aryepiglottic fold and obstructed most of the laryngeal cavity (Fig. 1A and B). The intraepithelial papillary capillary loop (IPCL) was not visible in narrow-band imaging (NBI) mode (Fig. 1C). Afterward, enhanced computed tomography (CT) showed a shadow of soft tissue density in the left supraglottic region with an indistinct border with surrounding tissues and uneven enhancement (Fig. 2A and B).

**Figure 3.**
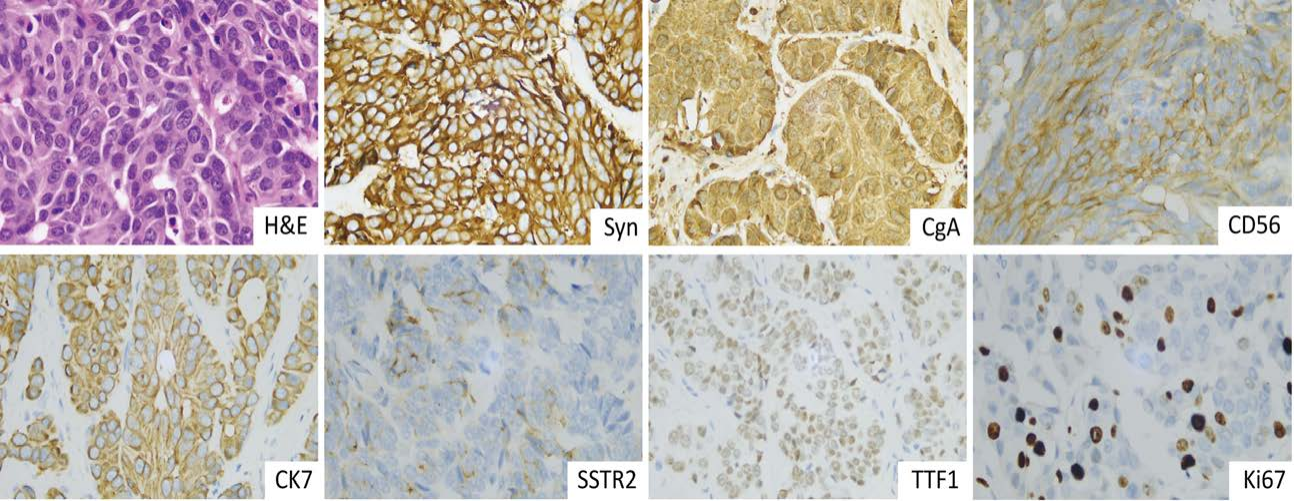
Hematoxylin and eosin stain showed the tumor is composed of epithelioid cells that are arranged in sheets, with rounded or oval nuclei and speckled nuclear chromatin (magnification × 400). Immunohistochemistry revealed positive expression of Syn, CgA, CD56, CK7, TTF-1, and a small amount of SSTR2 (×400). Proliferative index Ki67 was 20% (×400).

During hospitalization, the patient experienced sudden loss of consciousness due to respiratory distress, resulting in a drop in oxygen saturation to 46%. An emergency bedside tracheotomy was performed. Subsequently, the mass was biopsied and the pathology result showed poorly differentiated adenocarcinoma. After the patient condition stabilized, a horizontal hemilaryngectomy and left neck dissection were performed. The excised mass measured 2.5 cm by 2.1 cm by 1.8 cm and had a slightly firm texture. Postoperative pathology revealed a laryngeal NET (G3), and metastasis occurred in 4 of the 18 lymph nodes in the left neck dissection. The immunohistochemical results showed positive expression of Syn, CD56, CgA, CK7, TTF-1, and a small amount of SSTR2, while negative expression of P63 was observed. Proliferative index Ki67 was 20% (Fig. 3). Written informed consent was obtained from his wife for all the above operations. At 4 weeks after the operation, the patient underwent 2 cycles of chemotherapy (cisplatin, etoposide) and radical radiotherapy (6000 cGy/30F). After a 1-year postoperative follow-up, physical examination and flexible fiberoptic laryngoscopy did not reveal any recurrence or metastasis (Fig. 1D).

## 3. Discussion

NENs are a heterogeneous group of tumors that originate from the neural ectoderm or epithelium and can occur anywhere in the body.^[7]^ They exhibit various histopathologic, molecular, and clinical features, making clinical diagnosis and treatment challenging.^[8]^ Therefore, it is common for NENs to have already metastasized by the time of diagnosis. According to literature reports, this can occur in up to 56% to 69% of cases.^[9,10]^ In the larynx, NENs are much rarer and have historically been classified using the diagnostic terminology of enteric and pulmonary NENs.^[11]^ In 2017, the World Health Organization (WHO) systematically classified and named the head and neck NENs as well-differentiated carcinoma G1; moderately-differentiated and poorly differentiated G3 with small cell NEC (SmCNEC) and large cell NEC (LCNEC).^[12]^ The corresponding 5-year survival rates are 100%, 52.8%, 19.3%, and 15.3%, respectively.^[11]^

Laryngeal NENs are frequently found in men aged 50 to 70, with a male-to-female ratio of approximately 3:1. These tumors are usually located in the supraglottis and are linked to a history of smoking.^[6,12]^ In the present study, we present a 67-year-old man with a clear history of smoking and the mass was located in the supraglottic region, which is consistent with previous literature. Patients with laryngeal NEN typically experience local symptoms related to the occupancy of the mass, such as a sore throat, hoarseness, and difficulty breathing. However, detecting the disease in its early stages is challenging because the supraglottic region is concealed. Therefore, patients usually seek medical attention when the mass has grown to a larger size. In this paper, the patient presented with obvious symptoms of breath-holding and loss of consciousness, and was revived after an emergency cricothyrotomy, which increased the risk and difficulty of clinical diagnosis and treatment.

NENs originate from neuroendocrine cells that secrete hormones and proteins (e.g., insulin, gastrin, vasoactive intestinal peptide), which can serve as biomarkers for NENs.^[13]^ However, no specific biomarkers have been identified for laryngeal NENs. In this report, the patient underwent preoperative flexible fiberoptic laryngoscopy, enhanced neck CT and local biopsy. The flexible fiberoptic laryngoscopies showed grayish-white cauliflower-like masses in white light mode, which was similar to laryngeal squamous cell carcinoma in appearance. However, IPCL was not observed in NBI mode, which may be a crucial feature in distinguishing NENs from squamous cell carcinomas. Enhanced CT showed a shadow of soft tissue density, indicating a probable malignancy, but a definitive diagnosis could not be confirmed. Since NENs can characteristically express cell-surface peptide hormone receptors, growth inhibitory receptor (SSTR)-based ^68^Ga-DOTA PET/CT shows definite advantages in the diagnosis and treatment of NENs.^[14]^ Zhi et al performed ^68^Ga-DOTA-SSTR PET/CT on 14 patients with head and neck paragangliomas before receiving treatment, and showed that it could identify primary and metastatic tumor and distinguish different subtypes based on quantification.^[15]^ Nevertheless, due to the low incidence of laryngeal NENs, there are limited studies on the application of this examination. In our report, the patient was unable to undergo ^68^Ga-DOTA-SSTR PET/CT due to technical limitations, which resulted in some disadvantages in the follow-up of this disease. The combination of morphology and immunohistochemistry remains the mainstay of diagnosis of NENs.^[5]^ Histologically, NETs are classified into highly differentiated endocrine tumors (NETs) and poorly differentiated NECs. NETs have small, well-differentiated cells with relatively homogeneous round to oval nuclei, and exhibit a “salt-and-pepper” appearance due to the numerous secretory granules they produce. NECs tumors have poorly differentiated cells that proliferate in a “sheetlike” pattern and express fewer neuroendocrine markers.^[7]^ The diagnosis of laryngeal NENs currently relies on several immunohistochemical markers, including synaptophysin (Syn), chromogranin-A (CgA), CD56, Ki-67, calcitonin, carcinoembryonic antigen (CEA), and thyroid transcription factor-1 (TTF1).^[11]^ Of these, Syn is the most sensitive for diagnosing NENs, while CgA is the most specific.^[8]^ Recently, INSM1 was shown to diagnose NENs with high sensitivity and specificity.^[8]^ Yuan et al performed immunohistochemical staining of INSM1, CgA, Syn, and CD56 in pathological samples of 25 laryngeal NENs and the results showed that INSM1 had a higher sensitivity (92%) and specificity (91%) compared to the other 3.^[16]^ Ki-67 as a proliferative marker, combined with mitotic counting and necrosis, can be used to grade laryngeal NENs. The applications of calcitonin, CEA, and TTF1 in head and neck tumors have primarily focused on thyroid tumors. However, these markers have also been reported in laryngeal NENs, and further exploration of their diagnostic significance is necessary.^[4,17,18]^ In this report, we performed preoperative mass biopsy on the patient, but the pathology initially diagnosed as an adenocarcinoma due to their morphological similarity. Then the patient underwent radical resection of the tumor, and postoperative pathology confirmed the diagnosis of laryngeal NET by immunohistochemical staining for Syn (+), CD56 (+), and CgA (+). The tumors were identified as G3 based on histological morphology, Ki-67, and SSTR2.

In the literature, laryngeal NENs are usually managed with surgery or chemoradiotherapy.^[6,19,20]^ However, due to their rarity, there is currently no established standard treatment protocol. The case of laryngeal NET in our report underwent radical surgery. After surgery, he received chemotherapy (cisplatin, etoposide) and radical radiotherapy (6000 cGy/30F). The patient showed no signs of recurrence at the 1-year postoperative follow-up and achieved complete remission. Becht et al reported a patient with subglottic laryngeal NET who received chemoradiotherapy and survived for 29 months after diagnosis.^[19]^ Sousa et al conducted a retrospective analysis of 15 patients with high-grade NEC of the larynx or hypopharynx, and the results showed that the median overall survival was 17.8 months.^[21]^ Therefore, long-term follow-up is still necessary to observe the treatment effect on the patient.

It is well known that laryngeal NENs have a high recurrence rate and can negatively impact prognosis. Therefore, new therapeutic strategies are necessary. NENs often express growth inhibitory receptors, making somatostatin analogs (SSA) recommended as the first-line therapy for patients with advanced NEN who have positive SSTR receptor-associated imaging. However, guidelines specify that SSA should not be used as an adjunctive therapy for patients with gastroenteropancreatic or lung NENs who have undergone radical surgery.^[22]^ Studies on SSA in laryngeal NENs have rarely been reported, and further verification of its therapeutic effects is necessary. Additionally, the literature reports the successful use of monoclonal antibodies targeting PD-L1 to treat cases of laryngeal NENs, but this still requires confirmation through large-scale clinical trials.^[23]^

## 4. Conclusions

Laryngeal NENs are a rare and heterogeneous group of tumors in the head and neck. Diagnosis is based on a combination of morphology and immunohistochemistry. Treatment mainly involves surgery and chemoradiotherapy, but there is no standardized protocol. Recurrence and metastasis significantly impact prognosis, highlighting the need for further exploration of personalized and precise diagnosis and treatment.

## Acknowledgments

We thank Guihui Zhang (a pathologist) for providing the pathology images for this report.

## Author contributions

**Conceptualization:** Longqing Ding, Jiahui Liu, Ying Guo.

**Resources:** Yaqi Wang, Yisong Yao, Xi Chen, Yakui Mou.

**Validation:** Xicheng Song.

**Writing – original draft:** Longqing Ding, Jiahui Liu, Ying Guo.

**Writing – review & editing:** Xicheng Song.
